# Early use of low-dose hydrocortisone can reduce in-hospital mortality in patients with septic shock: A systematic review and meta-analysis

**DOI:** 10.1097/MD.0000000000040635

**Published:** 2024-11-29

**Authors:** Binglin Song, Xiangde Zheng, Kangrui Fu, Chun Liu

**Affiliations:** a North Sichuan Medical College, Sichuan, China; b Emergency Department, Dazhou Central Hospital, Sichuan, China.

**Keywords:** early stage, hydrocortisone, late stage, septic shock, timing

## Abstract

**Background:**

This study aimed to assess the impact of the timing of low-dose hydrocortisone adjuvant therapy initiation on clinical outcomes in patients with septic shock by a systematic review and meta-analysis.

**Methods:**

We conducted a comprehensive search of all randomized controlled trials (RCTs) and cohort studies available in the PubMed, Web of Science, and Embase databases. The search included articles published from the founding of these databases until August 1, 2024. The purpose of the search was to compare the results of initiating low-dose hydrocortisone (HC) adjuvant therapy at different time periods. The main reported results included short-term mortality (ICU mortality and hospital mortality) as key outcomes, and secondary outcomes such as the rate of renal replacement treatment continuous renal replacement therapy (CRRT), length of stay in the intensive care unit (ICU), and rate of shock reversal.

**Results:**

Seven trials, with a total of 3063 patients, were included. The main finding of this meta-analysis indicates that the early treatment group, which received low-dose hydrocortisone, had a lower ICU mortality rate compared to the late treatment group. Additionally, the hospital mortality rate in the early treatment group was lower than that in the late treatment group. There was a correlation between the timing of beginning of HC and the short-term mortality of patients with septic shock. The secondary findings indicated that there were no notable disparities in the rates of CRRT, the rate of reversing shock, and the duration of stay in the ICU.

**Conclusion:**

Administering low doses of HC early on can decrease the risk of death in septic shock patients in the short-term mortality. There were no substantial disparities observed in the rate of CRRT, the rate of reversal of shock, and the duration of stay in the ICU. Additional extensive RCTs are required to validate this conclusion.

## 1. Introduction

Based on literature data, the estimated global fatality rate for severely ill patients with septic shock is around 40%.^[1–3]^ The current approach to treating septic Huk involves promptly administering fluids, using vasoactive medications, and prescribing antibiotics. Nevertheless, the extended utilization of vasoactive medications can result in a lengthened duration of hospitalization and heightened death rates.^[3–6]^ The Exercise Guidelines for Surviving Sepsis suggest using HC adjuvant therapy for individuals with severe symptoms who need a norepinephrine dose of 0.25 mg/kg/min or higher for more than 4 hours.^[6]^ The utilization of glucocorticoids in individuals experiencing septic shock may be traced back to the 1950s. The initial modest randomized controlled studies conducted by Hahn, Weitzman, and other researchers yielded contradictory outcomes.^[7,8]^ In 1987, Bone et al showed that administering high dosages of glucocorticoids to patients with severe sepsis and septic shock could lead to an elevated rate of mortality during their hospital stay.^[9,10]^ In 2002, Annane et al conducted a research which demonstrated that a 7-day regimen of low-dose hydrocortisone (HC) was effective in decreasing mortality rates.^[11]^ In 2004, the Surviving Sepsis Campaign (SSC) guideline provided recommendations for the administration of glucocorticoids for patients diagnosed with septic shock.^[12]^ In the studies of Sprung et al^[13]^ and Annane et al,^[11]^ 2 Sebille trials demonstrated that the use of hydrocortisone (HC) did not result in a decrease in the death rate of patients with septic shock.^[11,13]^ As a result, the guidelines from the SSC were modified from a “recommended” status in 2004 to a continued “recommended” status in 2008. In 2011, Annane, D. conducted a study along with Renault, A. et al which shown that HC-assisted treatment of sepsis resulted in a reduction in 90-day mortality to 43% compared to 49%.^[14]^ In 2010, Fang, F. also conducted a related study. A recent small-scale randomized controlled trial (COIITSS) conducted by et al demonstrated that the use of HC may lead to a decrease in 28-day mortality.^[15,16]^ Consequently, the 2021 SSC guidelines have updated their recommendation about the use of HC in patients with septic shock, now considering it a weak recommendation.^[2]^ A meta-analysis conducted in May 2023 examined the effects of low-dose hydrocortisone (HC) on persons with septic shock. The investigation found that HC may lead to an increased risk of mortality, with a relative risk (RR) of 0.86 for 90-day mortality.^[17]^Based on many research, our analysis determined that the use of low-dose hydrocortisone (HC) as an additional treatment may decrease the likelihood of death during a hospital stay for patients with septic shock. However, there is no specific information available regarding when to start HC therapy. We performed a meta-analysis by gathering data from published randomized controlled trials (RCTs) and cohort studies to evaluate the effects of starting low-doses of hydrocortisone (HC) at various time intervals on the clinical outcomes of patients with septic shock.

## 2. Methods

This investigation utilized the PRISMA procedures (PRISMA 2020), which are a set of systematic review and meta-analysis guidelines.^[18]^ This study constitutes a comprehensive examination of existing literature and therefore does not necessitate the acquisition of ethical approval. The protocol for this systematic review has been officially filed at INPLASY with the registration number 202480070.

### 2.1. Search strategy

**Figure 1.**
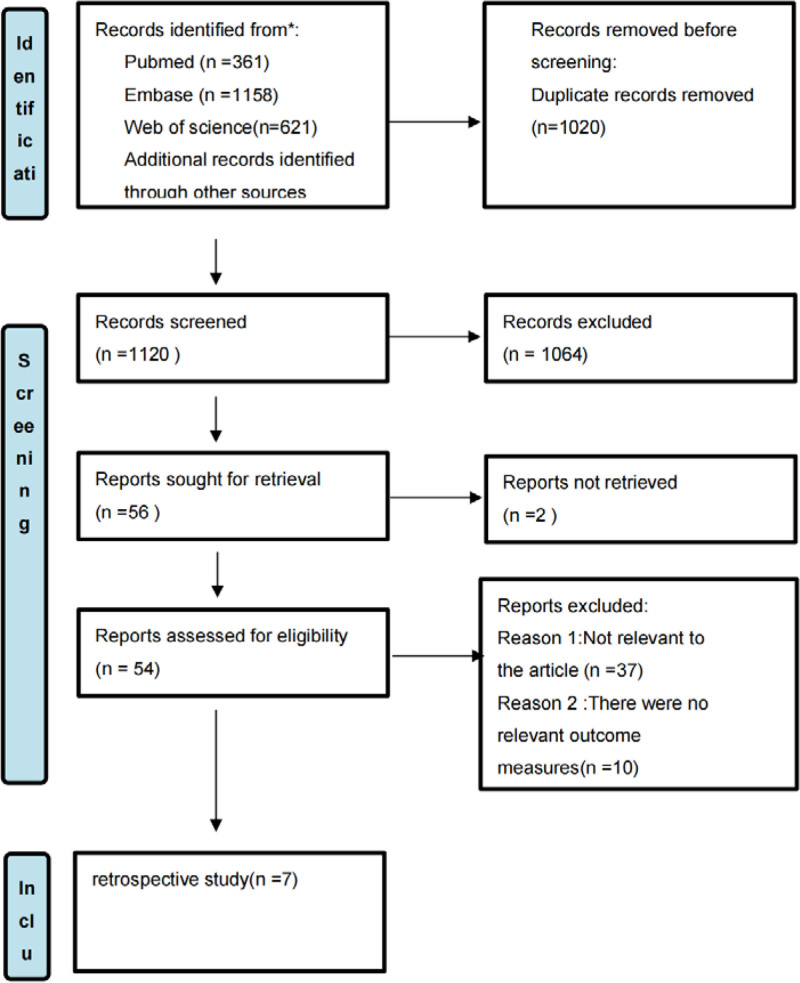
Document inclusion flow chart (All of the studies are non-randomized trials, of the included studies, 3 were from USA, 1 was from Greece, 1 was from Saudi Arabia, 1 was from Israel, and 1 was in Korean. A total of 3063 patients were involved, the sample sizes of 2 studies are more than 500, all study’s has a sample size of data > 100).

We conducted a comprehensive search in PubMed, Web of Science, and Embase databases for all publications published from the inception of the databases until August 1, 2024. The search was performed using the terms “septic shock,” “shock,” “hydrocortisone,” “timing,” “early,” and “late.” We made minor modifications to the search results to accommodate various database specifications. Additionally, we examined the author’s profile and reference lists to identify pertinent review articles. Figure 1 displays the flow chart of the search approach. A detailed search strategy is provided in the (Appendix 1, Supplemental Digital Content, http://links.lww.com/MD/O21).

### 2.2. Type of result measurement

The main measure of interest was the death rate in the short-term, specifically within the intensive care unit (ICU) and during the hospital stay. The secondary outcomes included the rate of renal replacement treatment, the length of stay in the ICU, and the rate of reversal of shock. The weighted average was computed by taking into account the patient count in each study.

### 2.3. Research options

The inclusion criteria for this study were as follows: RCTs, prospective and retrospective cohort studies; adult patients with septic shock who were over 18 years old. Septic shock was defined according to the current Third International Consensus Definition of Sepsis and Septic Shock (Sepsis 3.0), which includes the presence of infection with organ dysfunction, use of vasoactive drugs, mean arterial pressure (MAP) below 65 mm Hg, and lactate level above 2 mmol/L.^[19]^ The study provided primary end measures, including ICU mortality and in-hospital mortality, as well as secondary outcomes such as the rate of renal replacement treatment, length of stay in the ICU, and the rate of shock reversal. Studies that did not have clear comparison results were excluded. Furthermore, we specifically omitted review papers and research works pertaining to pediatrics or animals.

### 2.4. Quality assessment

The quality was independently assessed by 2 reviewers, Chun Liu and Binglin Song. The study quality was assessed using the randomized controlled trial method^[20]^ developed by The Cochrane Collaboration. The specific factors that mitigate bias in RCTs include: randomization of the sequence to reduce selection bias; concealing the assignment to reduce selection bias; blinding of both researchers and participants to reduce performance bias; blinding of outcome evaluators to reduce tabulator bias; complete reporting of data without arbitrary exclusion of patients; and blind reporting of data without arbitrary exclusion of patients. The minimum loss bias, selective reporting bias, and other forms of prejudice were followed up. Green, yellow, and red represent satisfied, unclear, and unsatisfied performance, respectively. The Newcastle-Ottawa Scale (NOS) was employed to assess the treatment in cohort studies.^[21]^ NOS allocates a maximum of 9 points according to the quality of population selection, comparability, and results in cohort studies. The definition of research quality was categorized as follows: poor (0–3), fair (4–6), and good (7–9). The Table 1 displays the quality of the cohort studies that are included.

**Table 1:** Quality assessment of included studies (Newcastle-Ottawa scale).

Study	Selection				Comparability	Outcome			Total score
	Representativeness of the exposed cohort	Selection of the non-exposed cohort	Ascertainment of exposure	Demonstration that outcome of interest was not present at the start of the study	Comparability of cohorts on the basis of the design or analysis	Assessment of outcome	Was follow up long enough for outcomes to occur	Adequacy of follow up of cohorts	
David 2021^[22]^	☆	☆	☆	☆	☆	☆	☆	☆	8
Chry 2014^[23]^	☆	☆	☆	☆	☆	☆	☆	☆	8
Moath 2023^[24]^	☆	☆	☆	☆	☆	☆	☆	☆	8
Ab 2024^[25]^	☆	☆	☆	☆	☆	☆	☆	☆	8
Gr 2020^[26]^	☆	☆	☆	☆	☆	☆	☆	☆	8
Luming 2023^[27]^	☆	☆	☆	☆	☆	☆	☆	☆	8
Hye 2012^[3]^	☆	☆	☆	☆	☆	☆	☆	☆	8

### 2.5. Data analysis

The program utilized for this task was Review Manager (RevMan) version 5.4. According to the Cochrane Manual of Systematic Review, the study measured in-hospital mortality and bleeding complications as dichotomous variables. The odds ratios (OR) with 95% confidence intervals (CI) were used to describe these variables. The weighted pooled odds ratio was then determined using the Mantel-Haenszel technique.^[28]^ The continuous variable, length of stay, is represented by the mean difference (MD) with a 95% confidence interval (95% CI). The outcome indicators of several publications were documented as the median and interquartile range (IQR), whereas the mean and standard deviation were computed using a calculator,^[29]^ taking into account the sample size. The I² test was employed to quantify statistical heterogeneity.^[30,31]^ When the value of I² is equal to 0, there is no heterogeneity present. When the value of I² is <50%, the research heterogeneity is considered to be minor, and the fixed-effect model is used for analysis.^[31]^ When the value of I² is >50%, the research heterogeneity is considered to be relatively significant, and the random effect model is used for analysis. Simultaneously, a sensitivity analysis was performed to demonstrate the stability of the findings. To mitigate bias, we performed subgroup analysis on RCTs and cohort studies. The findings were consolidated in Figure 1, employing bilateral testing where statistical significance was determined by a *P*-value of <.05.

## 3. Results

A total of 2140 literatures were first reviewed, encompassing the specific process of literature screening and the fundamental characteristics of the included literatures. 1020 redundant literatures were eliminated, leaving 1120 literatures. Upon reviewing the titles and abstracts, a total of 54 articles were initially selected. Ultimately, the entire text was thoroughly examined, and any literature that lacked a direct connection and relevant data was eliminated. Consequently, only 7 literatures were ultimately chosen, as depicted in Figure 1. Table 2 displays the fundamental details of the referenced literature. Seven cohort studies, as indicated in Table 2, involved a total of 3063 patients.^[3,22–27]^ The outcome markers of these studies may be seen in Table 3. From 2012 to 2024, a total of 7 suitable studies were published. Out of these, 5 were published after 2020. The studies included 3 from the United States, 1 from Korea, 1 from Israel, 1 from Greece, and 1 from an Arab country. All of these studies were conducted at a single center. The meta-analysis includes definitions of early and late hydrocortisone use, which can be seen in Table 4.

**Table 2:** Basic information included in the study.

Study	Study design	Country	Time period	Patients	Dose	Age	SOFA	MAP at vasopressor initiation (mm Hg)
David 2021^[22]^	Review	USA	2014.7.1 to 2019.8.31	99/99	≤300 mg/d HC	69.7 (61–77.2) versus 68.4 (60.5–77.8)	12 (10–15)/12 (9–15)	60 (52–64)/63 (57–68)
Chry 2014^[23]^	Non-randomized prospective longitudinal study	Greece	2009.1 to 2011.1 2012.12 to 2013.2	46/124	50 mg, q6h/d,	65.2 ± 19.7 versus 62.8 ± 16.9	11.5 ± 3.8/10.7 ± 3.6	NR
Moath 2023^[24]^	Non-randomized prospective longitudinal study	Saudi Arabia	2022.2 to 2022.8	46/35	NR	63 (17) versus 56 (18)	3 (2–3)/3 (3–3)	NR
Ab 2024^[25]^	Review	USA	2017.1.1 to 2019.12.31	62/60	HC 200 to 300/d	66 (60–72)/65 (61–72)	11 (9–13)/11 (9–14)	60 (54–63)/61 (57–63)
Gr 2020^[26]^	Review	USA	2011.1 to 2017.11	1058/412	Intermittent 50 mg or 100 mg	62 ± 15/60 ± 14	NR	66.3 ± 14.8/66.6 ± 13.9
Luming 2023^[27]^	Review	Israel	2008 to 2019	553/291	NR	66.0 (57.0–76.0)/67.0 (58.0–76.5)	4.0 (3.0–6.0)/4.0 (3.0–7.0)	NR
Hye 2012^[3]^	Review	Korea	2008.1 to 2009.12	66/112	≤300 mg/d HC	67 (55–71)/65 (53–71)	11 (9–13)/11 (9–14)	NR

Abbreviations: HC = hydrocortisone MAP = mean arterial pressure.

**Table 3:** Outcome data of the included literatures.

Study	Patients	In-hospital mortality	In-ICU mortality	Need for renal replacement therapy, n (%)	Reversal of shock	ICU length of stay, d
David 2021^[22]^	99/99	42 (42.4%) versus 48 (48.5%)		23 (23.2%) versus 24 (24.2%)		3.6 (1.8–9.2) versus 5.1 (3–9.9)
Chry 2014^[23]^	46/124	15 (33.3%) versus 68 (53.3%)				
Moath 2023^[24]^	46/35		25 (54.3%) versus 19 (54.3%)	13 (28.3%) versus 11 (31.4%)	35 (76.1%)/24 (68.6%)	17 versus 20
Ab 2024^[25]^	62/60	35 (56.4%) versus 35 (58.3%)	34 (54.8%)/34 (56.6%)	20 (32.2%)/13 (21.6%)		5 (4–8)/7.5 (4–12)
Gr 2020 (24 h)^[26]^	1058/412	533 (50.4%) versus 235 (57.0%)	472 (44.6%) versus 199 (48.3%)			ICU 10.1 ± 13.0/13.7 ± 12.9
Luming 2023^[27]^	553/291	228 (41.2%) versus 173 (59.5%)				9.4 (4.2–18.4)/9.5 (3.3–20.1)
Hye 2012^[3]^	66/112	29 (44%) versus 75 (67%)	21 (32%)/55 (49%)	19 (29%)/39 (35%)	50 (76%)/70 (63%)	4 (3–8)/7 (4–12)
Gr 2020 (6 h)^[26]^	567/903		275 (48.5%)/493 (54.5%)	244 (43.0)/427 (47.2)		
Gr 2020 (12 h)^[26]^	798/672		396 (49.6)/372 (55.3)	352 (44.1)/319 (47.4)		

**Table 4:** Criteria for early and late stage definitions in included studies.

Study	Early group	Later group
David 2021^[22]^	<12 h after vasopressors initiation	>12 h after vasopressors initiation
Chry 2014^[23]^	<9 h after vasopressors initiation	>9 h after vasopressors initiation
Moath 2023^[24]^	<3 h after diagnosis of septic shock	>6 h after diagnosis of septic shock
Ab 2024^[25]^	<12 h after vasopressors initiation	>12 h after vasopressors initiation
Gr 2020^[26]^	<24 h after diagnosis of septic shock	>24 h after diagnosis of septic shock
Gr 2020^[26]^	<6 h after diagnosis of septic shock	>6 h after diagnosis of septic shock
Gr 2020^[26]^	<12 h after diagnosis of septic shock	>12 h after diagnosis of septic shock
Luming 2023^[27]^	<12 h after vasopressors initiation	>12 h after vasopressors initiation
Hye 2012^[3]^	<6 h after diagnosis of septic shock	>6 h after diagnosis of septic shock

### 3.1. In-ICU mortality

**Figure 2.**
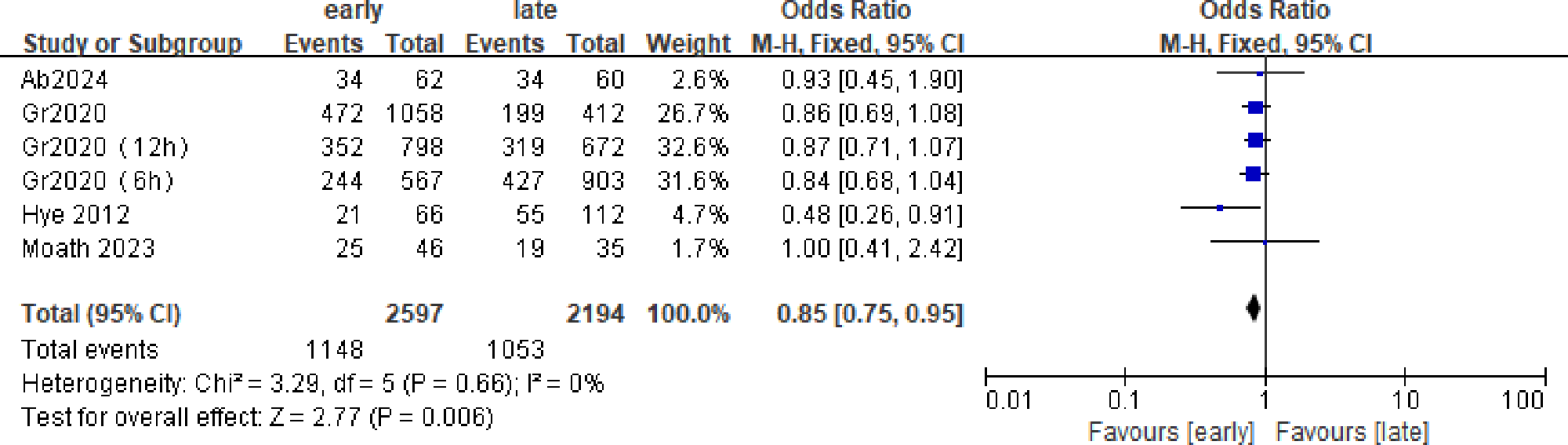
Forest plot comparing the effects of early and late use of hydrocortisone on the in-ICU mortality.

Of 1851 patients enrolled In 5 studies, the ICU mortality rate was approximately 46%. (Early group 552/1232, late group 307/619). The ICU mortality in the early group was lower than that in the late group (odds ratio [OR] = 0.85; 95% CI = 0.75–0.95; *P* = .006; χ^2^ = 2.77; *I*^2^ = 0%) (Fig. 2).

### 3.2. In-hospital mortality

**Figure 3.**
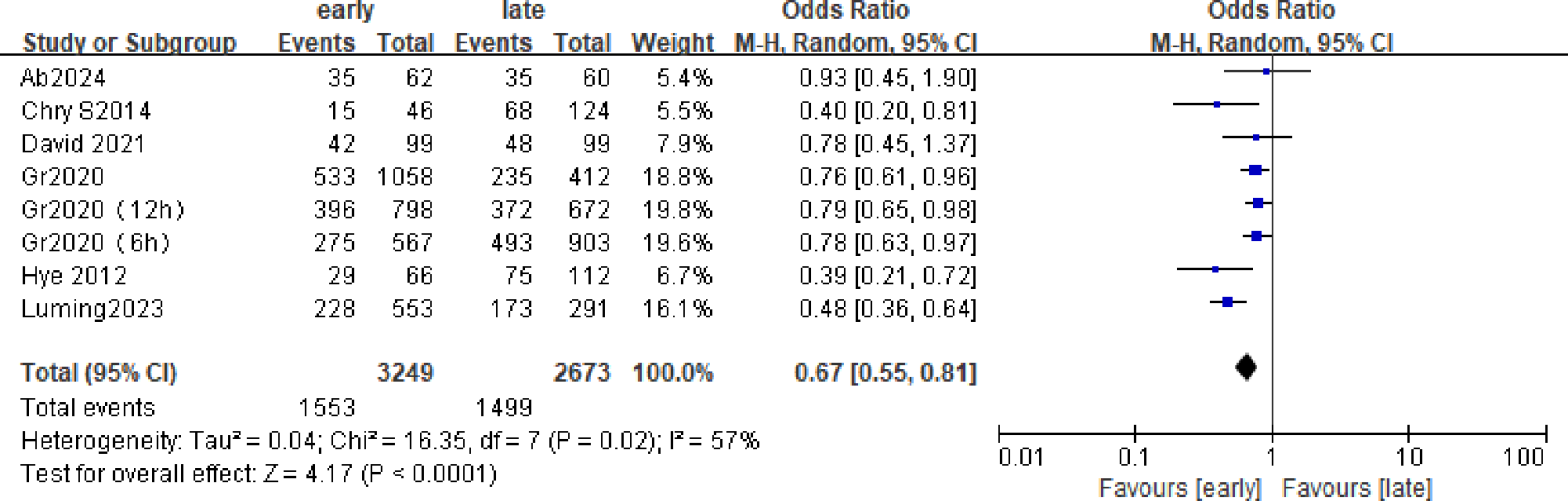
Forest plot comparing the effects of early and late use of hydrocortisone on the in-hospital mortality.

**Figure 4.**
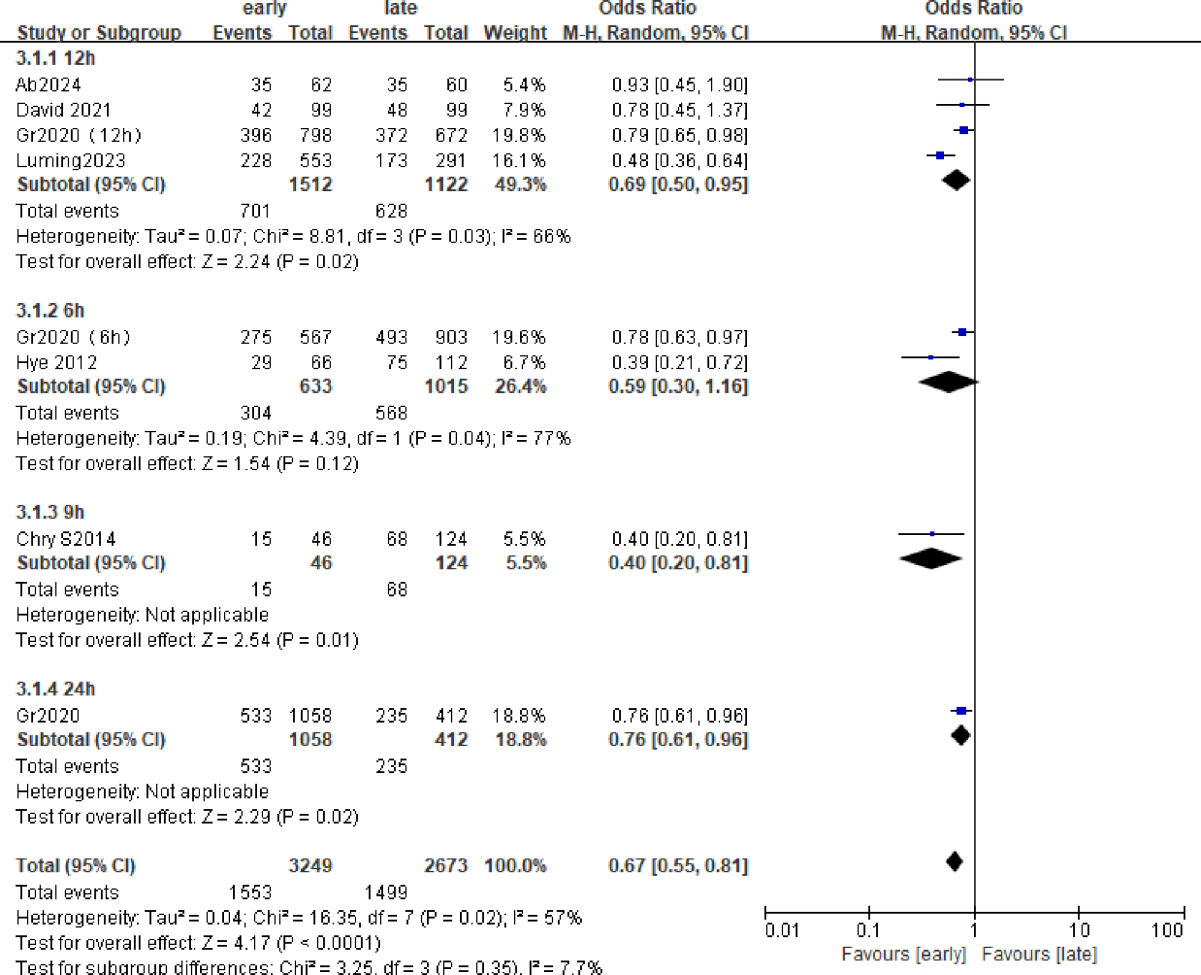
Forest plot comparing the effects of early and late use of hydrocortisone on the in-hospital mortality subgroup.

The 6 included studies were analyzed to assess in-hospital mortality. A total of 2982 patients were included, and the death rate during hospitalization was about 50%. (Early group 882/1884 and late group 634/1098), the hospital mortality rate of early group was lower than that of late group (odds ratio [OR] = 0.67; 95% CI = 0.55–0.81; *P* < .0001; χ^2^ = 2.77; *I*^2^ = 57%) (Fig. 3). Four subgroups (6, 9, 12h) were analyzed according to the start time of hydrocortisone use. As shown in Figure 4, *I*^2^ of all subgroups was >50%, so the random effects model was selected, and the data showed that group 12 h ([OR] = 0.69; 95% CI = 0.50–0.95; *P* = .02; χ^2^ = 2.24; *I*^2^ = 66%), 6 h group ([OR] = 0.59; 95% CI = 0.30–1.16; *P* = .12; χ^2^ = 1.54; *I*^2^ = 77%). Early use of hydrocortisone was associated with a reduction in in-hospital mortality (pooled OR = 0.67, 95% CI: 0.55–0.81, *P* < .0001) (Fig. 4).

### 3.3. Need for renal replacement therapy, n (%)

**Figure 5.**
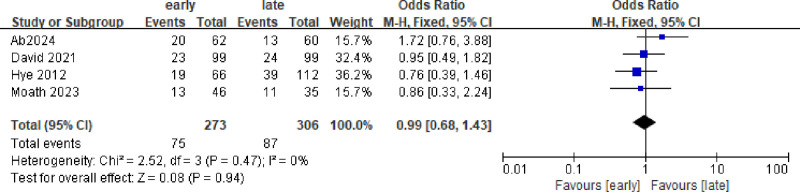
Forest plot comparing the effects of early and late use of hydrocortisone on the need for renal replacement therapy, n (%).

A total of 4 studies with 579 patients were included, and the results showed no significant difference Figure 5.

### 3.4. Shock reversal rate

**Figure 6.**
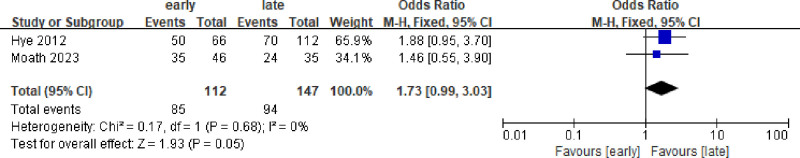
Forest plot comparing the effects of early and late use of hydrocortisone on the shock reversal.

A total of 2 studies with 2599 patients were included, and results showed no significant difference Figure 6.

### 3.5. ICU length of stay, days

**Figure 7.**
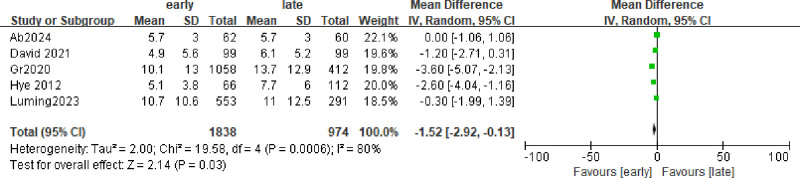
Forest plot comparing the effects of early and late use of hydrocortisone on the ICU length of stay, days.

The data were expressed as mean ± SD, and part of the data was median ± IQR. We converted the data through the formula,^[32]^ and a total of 5 studies^[17,24,25]^ were included, showing significant heterogeneity (Fig. 4; I² = 80%, *P* = .03). The data showed that the ICU time in the early group was longer than that in the late group (RR = −1.52; 95% CI = −2.92 to 0.13; *P* = .03), and *I*^2^ = 80% (Fig. 7).

**Figure 8.**
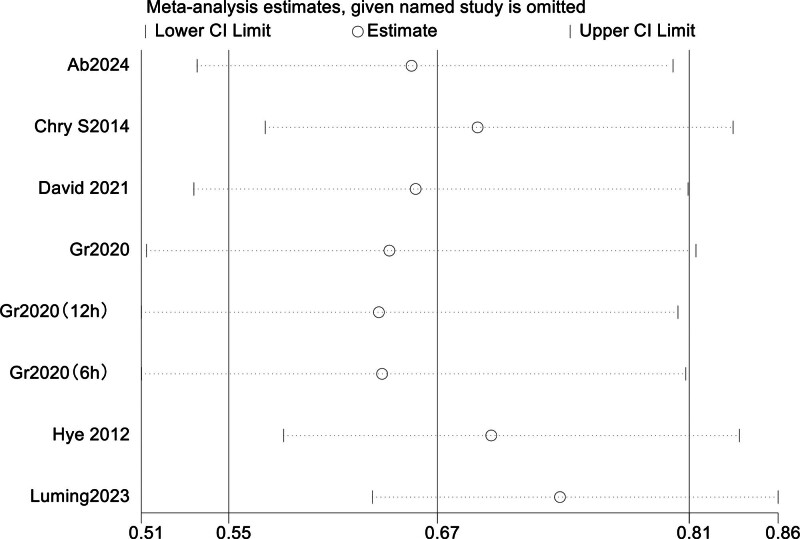
In-hospital mortality sensitivity analysis.

**Figure 9.**
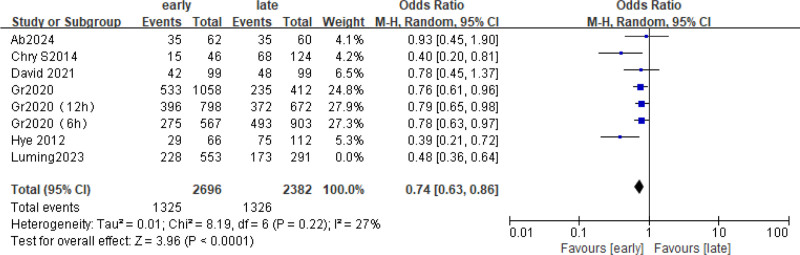
Forest map of hospital mortality after removing high heterogeneity from Luming 2023.

Sources of heterogeneity were further identified with sensitivity analyses of highly heterogeneous outcomes, which are shown in a forest plot of in-hospital mortality (I² = 57%, *P* < .0001) (Fig. 8). In the forest chart of inpatient mortality, references that deviated significantly from the median line were removed one by one. After Luming 2023^[27]^ was removed, heterogeneity was significantly reduced (*I*^2^ = 27% vs *I*^2^ = 57%). Under the fixed-effect model (OR = 0.74, 95% CI = 0.63–0.86; *P* < .0001). *I*^2^ = 27%, so it is determined that this article may be the source of heterogeneity. The forest map is shown in Figure 9.

## 4. Discussion

This study conducted a systematic review and meta-analysis of 7 studies, involving a total of 3063 patients, to examine the timing of hydrocortisone medication initiation in patients with septic shock. The study revealed an ICU death rate of approximately 46%, with a decreased ICU mortality rate observed in the early group compared to the late group. The death rate within the hospital was approximately 50%, and the mortality rate in the early group was reduced compared to the late group. The 6h group had the lowest mortality rate as the cutoff point ([OR] = 0.59), followed by the 12h group ([OR] = 0.69). The precise mechanism by which the early administration of a low dose of hydrocortisone improves survival is yet unknown. The explanation may be influenced by the regulation of endogenous cortisol metabolism, which is reduced in critically ill patients. This reduction can hinder lymphocyte resistance to the anti-inflammatory effects of glucocorticoids and increase sensitivity to vasopressors due to hydrocortisone or the mineralized corticosteroid properties of hydrocortisone.^[33–36]^ A study conducted in 2002 discovered that individuals who used HC in its early stages exhibited reduced production of TNF-α,^[11]^ a cytokine that plays a crucial role in the body’s response to infection and is linked to the vasodilation observed in sepsis. The immunomodulatory properties of hydrocortisone are anticipated to play a crucial role in the positive effects of starting treatment early. Several studies have determined that administering hydrocortisone early on helps expedite the reversal of shock and decrease the need for vasopressors. The study conducted by David in 2021^[22]^ shown that the early administration of hydrocortisone can considerably reduce the duration of vasopressors usage (40.7 hours vs 60.6 hours, *P* = .0002). Moath 2023^[24]^ found that the time required to stop vasopressor drugs in the early and late groups was 25 hours and 37 hours, respectively (*P* = .009), and more patients could achieve shock reversal (35 cases vs 24 cases). A study of Ab 2024^[25]^ with a total of 122 patients included in the analysis showed that early use of HC reversed shock in a shorter time compared to the advanced group (34 hours vs 65 hours; *P* = .012). Chry 2014^[23]^ found that compared with the late group, the time of vasoactive drug withdrawal in the early group was earlier than that in the late group, and the median time of drug withdrawal in the early start group was 4 days. In the advanced group, it was extended to 15 days (*P* < .0001), but we did not extract valid data for further analysis. The current guidelines from the SSC indicate norepinephrine as the initial choice of vasopressor for septic shock.^[2]^ Nevertheless, there is substantial evidence indicating that administering high doses of norepinephrine to individuals with septic shock carries specific risks.^[37]^ It is important to take into account the potential dangers of vasophosphoric therapy. Studies have demonstrated that higher doses of catecholamines, particularly those above 0.5 to 1 mcg/kg/min, can raise the likelihood of experiencing tachyarrhythmia, stress cardiomyopathies, and cardiotoxicity. These conditions are known to be linked to increased mortality in cases of septic shock.^[38–41]^ This meta-analysis indicates that initiating HC early can lead to several important benefits, including faster attainment and maintenance of adequate perfusion pressure, prevention of organ dysfunction and its progression, shorter duration and lower dosage of catecholamines, and additional reduction in mortality for patients with septic shock. Corticosteroids are administered to treat septic shock by suppressing nuclear factor KB, which leads to a decrease in interleukin (I) -1, IL-6, tumor necrosis factor (TNF)-α, IL-8, TNF receptor 1 and 2.^[42]^ Furthermore, HC suppresses the activity of nitric oxide (NO) synthase and hinders the vasodilation caused by sepsis that is mediated by NO.^[43]^ Exogenous corticosteroids decrease the levels of endogenous cortisol, which helps to relieve the adrenal instability that contributes to hemodynamic instability in septic shock.^[44]^

The discussion regarding the use of glucocorticoids in the treatment of septic shock has been ongoing for fifty years. There is disagreement regarding the advantages, such as the length of shock, the need for vasopressors, and mortality rates, which must be balanced against the negative effects, such as infection, high blood sugar levels, and high sodium levels.^[45,46]^ Increasing data suggests that the use of HC decreases the likelihood of death during a hospital stay. A comprehensive study conducted in 2019, which examined 37 randomized controlled studies, demonstrated that HC significantly reduces mortality within 28 days compared to a placebo. Annane et al conducted a meta-analysis that included 61 RCTs with a total of 12,192 participants. The data demonstrated that hydrocortisone (HC) was advantageous in promoting recovery from septic shock.^[15,16]^ On the other hand, a different comprehensive evaluation and statistical analysis that encompassed 22 RCTs (n = 7297) revealed that there was no variation in mortality rates among patients with septic shock. However, there was a decrease in the duration of shock, the need for mechanical ventilation, and the length of hospital stay when low-dose corticosteroids were administered.^[47]^ These studies specifically examined the most effective dosage and method of administering HC for treating septic shock, but did not specifically investigate the time of when it should be administer ed. We conducted the initial review of all studies on the timing of hydrocortisone initiation. However, there is no consistent definition for the terms “early” or “late” initiation of corticosteroids in patients with septic shock. The 7 studies included in our meta-analysis used different definitions for the early and late groups. We provide a (Table 1) that demonstrates the respective definitions of early and late groups for each author. Additional clinical trials are required to establish the efficacy of hydrocortisone in reducing patient mortality. Specifically, investigations with time intervals of 3 hours, 6 hours, 9 hours, and 12 hours are needed to strengthen the result. Furthermore, more data is necessary to support these findings. Analyzed further were the effects of the timing of glucocorticoid initiation on the rate of initiating continuous renal replacement therapy (CRRT) and the length of ventilator use.

## 5. Limitations

Initially, it is important to note that the 7 research included in the analysis employed varying starting time points, and there was an insufficient number of studies conducted at the same time point to facilitate further analysis. Furthermore, several outcome indicators in the studies included were reported using the median, such as the duration of vasopressors use, the time taken to achieve the goal MAP, or the quantity of intravenous infusion within a 6-hour period. These indicators could not be assessed and compared. Furthermore, there is limited evidence from the included studies regarding the occurrence of organ dysfunction as a significant clinical outcome. Furthermore, it is worth noting that all of the research included in the analysis were cohort studies.

## 6. Conclusion

Administering hydrocortisone (HC) to patients with septic shock at an early stage can decrease the mortality rates both in the ICU and during the hospital stay. There were no significant differences seen between the 2 groups in terms of the duration of stay in the ICU, the rate of dialysis, and the rate of reversal of shock. These findings indicate that it may be beneficial to administer hydrocortisone promptly and with greater intensity as a component of the early treatment for septic shock. Nevertheless, additional extensive RCTs are required to validate these findings.

## Author contributions

**Conceptualization:** Binglin Song.

**Data curation:** Binglin Song.

**Formal analysis:** Binglin Song.

**Funding acquisition:** Chun Liu.

**Investigation:** Chun Liu.

**Methodology:** Chun Liu.

**Project administration:** Chun Liu.

**Resources:** Xiangde Zheng.

**Software:** Xiangde Zheng.

**Supervision:** Xiangde Zheng.

**Validation:** Xiangde Zheng, Kangrui Fu.

**Visualization:** Kangrui Fu.

**Writing – original draft:** Kangrui Fu.

**Writing – review & editing:** Kangrui Fu.

## Supplementary Material


