# Supplementary material for: Early use of low-dose hydrocortisone can reduce in-hospital mortality in patients with septic shock: A systematic review and meta-analysis
Source: Medicine (Baltimore). 2024 Nov 29;103(48):e40635. doi: 10.1097/MD.0000000000040635 (PMC11608750; doi:10.1097/MD.0000000000040635)
Supplement: Supplementary file 1 [file medi-103-e40635-s001.doc]

**Appendix 1**

Pubmed 361

("early"[All Fields] OR "late"[All Fields] OR ("time"[MeSH Terms] OR "time"[All Fields]) OR ("timely"[All Fields] OR "timing"[All Fields] OR "timings"[All Fields])) AND ((((("progesterone"[MeSH Terms] OR "progesterone"[All Fields] OR "pregn 4 ene 3 20 dione"[All Fields]) AND "11 17 21 trihydroxy"[All Fields]) AND "11beta"[All Fields]) OR ("cortisol s"[All Fields] OR "cortisole"[All Fields] OR "Hydrocortisone"[MeSH Terms] OR "Hydrocortisone"[All Fields] OR "cortisol"[All Fields] OR "cortisols"[All Fields]) OR ("Hydrocortisone"[MeSH Terms] OR "Hydrocortisone"[All Fields]) OR ("Hydrocortisone"[MeSH Terms] OR "Hydrocortisone"[All Fields] OR "cortril"[All Fields]) OR ((("Hydrocortisone"[MeSH Terms] OR "Hydrocortisone"[All Fields] OR "hydrocortisones"[All Fields]) AND ("9"[All Fields] AND "beta 10"[All Fields] AND "alpha 11"[All Fields] AND ("alpha"[All Fields] OR "alpha s"[All Fields] OR "alphas"[All Fields]))) AND ("isomerism"[MeSH Terms] OR "isomerism"[All Fields] OR "isomer"[All Fields] OR "isomers"[All Fields])) OR ((("Hydrocortisone"[MeSH Terms] OR "Hydrocortisone"[All Fields] OR "hydrocortisones"[All Fields]) AND ("11"[All Fields] AND ("alpha"[All Fields] OR "alpha s"[All Fields] OR "alphas"[All Fields]))) AND ("isomerism"[MeSH Terms] OR "isomerism"[All Fields] OR "isomer"[All Fields] OR "isomers"[All Fields])) OR ("Hydrocortisone"[MeSH Terms] OR "Hydrocortisone"[All Fields] OR "epicortisol"[All Fields]) OR ("Hydrocortisone"[MeSH Terms] OR "Hydrocortisone"[All Fields] OR "11 epicortisol"[All Fields]) OR ("Hydrocortisone"[MeSH Terms] OR "Hydrocortisone"[All Fields] OR "cortef"[All Fields] OR "hydrocortisones"[All Fields]) OR "Hydrocortisone"[MeSH Terms]) AND ("shock, septic"[MeSH Terms] OR ("shock"[All Fields] AND "septic"[All Fields]) OR "septic shock"[All Fields] OR ("septic"[All Fields] AND "shock"[All Fields]) OR ("shock, septic"[MeSH Terms] OR ("shock"[All Fields] AND "septic"[All Fields]) OR "septic shock"[All Fields] OR ("shock"[All Fields] AND "endotoxic"[All Fields]) OR "shock endotoxic"[All Fields]) OR ("shock, septic"[MeSH Terms] OR ("shock"[All Fields] AND "septic"[All Fields]) OR "septic shock"[All Fields] OR ("endotoxin"[All Fields] AND "shock"[All Fields]) OR "endotoxin shock"[All Fields]) OR ("shock, septic"[MeSH Terms] OR ("shock"[All Fields] AND "septic"[All Fields]) OR "septic shock"[All Fields] OR ("endotoxin"[All Fields] AND "shocks"[All Fields]) OR "endotoxin shocks"[All Fields]) OR ("shock, septic"[MeSH Terms] OR ("shock"[All Fields] AND "septic"[All Fields]) OR "septic shock"[All Fields] OR ("shock"[All Fields] AND "endotoxin"[All Fields]) OR "shock endotoxin"[All Fields]) OR ("shock, septic"[MeSH Terms] OR ("shock"[All Fields] AND "septic"[All Fields]) OR "septic shock"[All Fields] OR ("shocks"[All Fields] AND "endotoxin"[All Fields])) OR ("shock, septic"[MeSH Terms] OR ("shock"[All Fields] AND "septic"[All Fields]) OR "septic shock"[All Fields] OR ("shock"[All Fields] AND "toxic"[All Fields]) OR "shock toxic"[All Fields]) OR ("shock, septic"[MeSH Terms] OR ("shock"[All Fields] AND "septic"[All Fields]) OR "septic shock"[All Fields] OR ("toxic"[All Fields] AND "shock"[All Fields]) OR "toxic shock"[All Fields]) OR ("shock, septic"[MeSH Terms] OR ("shock"[All Fields] AND "septic"[All Fields]) OR "septic shock"[All Fields] OR ("toxic"[All Fields] AND "shock"[All Fields] AND "syndrome"[All Fields]) OR "toxic shock syndrome"[All Fields]) OR ("shock, septic"[MeSH Terms] OR ("shock"[All Fields] AND "septic"[All Fields]) OR "septic shock"[All Fields] OR ("shock"[All Fields] AND "syndrome"[All Fields] AND "toxic"[All Fields]) OR "shock syndrome toxic"[All Fields]) OR ("shock, septic"[MeSH Terms] OR ("shock"[All Fields] AND "septic"[All Fields]) OR "septic shock"[All Fields] OR ("toxic"[All Fields] AND "shock"[All Fields] AND "syndromes"[All Fields]) OR "toxic shock syndromes"[All Fields]) OR "shock, septic"[MeSH Terms]))

Web of science 621

**1.(((((((((TS=(hydrocortisone)) OR TS=(Pregn-4-ene-3,20-dione, 11,17,21-trihydroxy-, (11beta)-)) OR TS=(Cortisol)) OR TS=(Cortifair)) OR TS=(Cortril)) OR TS=(Hydrocortisone, (9 beta,10 alpha,11 alpha)-Isomer)) OR TS=(Hydrocortisone, (11 alpha)-Isomer)) OR TS=(Epicortisol)) OR TS=(11-Epicortisol)) OR TS=(Cortef)**

**2.(((((((((TS=(Septic Shock)) OR TS=(Shock, Endotoxic)) OR TS=(Shock, Endotoxic)) OR TS=(Endotoxin Shocks)) OR TS=(Endotoxin Shocks)) OR TS=(Shocks, Endotoxin)) OR TS=(Shock, Toxic)) OR TS=(Toxic Shock)) OR TS=(Toxic Shock Syndrome)) OR TS=(Shock Syndrome, Toxic)**

**3.(((TS=(early)) OR TS=(late)) OR TS=(time)) OR TS=(timing)**

**#1 AND #2AND #3**

**Embase 1158**

**'septic shock'**/exp OR **'septic shock'** OR (**shock,** AND **endotoxic**) OR (**endotoxin** AND **shock**) OR (**endotoxin** AND **shocks**) OR (**shock,** AND **endotoxin**) OR (**shocks,** AND **endotoxin**) OR (**shock,** AND **toxic**) OR (**toxic** AND **shock**) OR **'toxic shock syndrome'** OR (**shock** AND **syndrome,** AND **toxic**) OR (**toxic** AND **shock** AND **syndromes**)

**'hydrocortisone'**/exp OR **hydrocortisone** OR **cortisol** OR **cortifair** OR **cortril** OR **epicortisol** OR **'11 epicortisol'** OR **cortef**

**early** OR **late** OR **time** OR **timing**

**#1 AND #2 AND #3**
